# Neuropeptide Y receptor Y2 (*npy2r*) deficiency reduces anxiety and increases food intake in Japanese medaka (*Oryzias latipes*)

**DOI:** 10.3389/fcell.2023.1273006

**Published:** 2023-11-07

**Authors:** Ke Lu, Xiaodan Jia, Jiaqi Wu, Qiuling Wang, Xu-Fang Liang

**Affiliations:** ^1^ College of Fisheries, Chinese Perch Research Center, Huazhong Agricultural University, Wuhan, China; ^2^ Engineering Research Center of Green Development for Conventional Aquatic Biological Industry in the Yangtze River Economic Belt, Ministry of Education, Wuhan, China

**Keywords:** NPY2R, Japanese medaka, knockout, food intake, anxiety

## Abstract

Neuropeptide Y receptor Y2 (*npy2r*) is an important receptor gene involved in anxiety and feeding regulation in mammals. Since NPY receptors have different receptor gene deletions in mammals and teleost fish, it is not clear whether *npy2r* has the similar function in fish as in mammals. In this study, we used the CRISPR/Cas9 system to establish *npy2r*-deficient medaka (*Oryzias latipes*). Unexpectedly, the deletion of *npy2r* resulted in the *npy2r* ^*+/−*^ medaka were all-male, therefore, *npy2r* homozygous mutant lines could not be established. The deletion of *npy2r* increased the food intake in medaka, and the expression levels of appetite stimulating genes (*agrp*, *npy*) increased significantly, while the expression levels of anorexia factors (*cck*, *pomc*) decreased significantly. Moreover, the absence of *npy2r* significantly increased the total length and body weight of medaka. The mirror test and open field test showed that *npy2r* ^*+/−*^ medaka improved sociability and reduced anxiety-like behavior, qRT-PCR analysis showed that the expression levels of anxiety related genes (*th1*, *th2*, *gr1*, *gr2*, and *mr*) in *npy2r* ^*+/−*^ medaka were significantly decreased. So far, this is the first *npy2r* gene knockout model established in fish and demonstrates that *npy2r* plays an important role in the regulation of reproduction, feeding and anxiety in fish.

## 1 Introduction

As an important neurotransmitter, Neuropeptide Y (NPY) was first identified in the pig brain (Tatemoto et al., 1982), and it is widely distributed in the central nervous system (Loh et al., 2015) and peripheral nervous system (Kuo et al., 2007) of vertebrates. By binding to different subtype receptors, NPY activates signal transduction pathways, which can regulate feeding (Matsuda et al., 2012), energy balance (Loh et al., 2015), anxiety (Kask et al., 2002; Shiozaki et a., 2020), circadian rhythm (Harrington et al., 2007) and other physiological processes. NPY receptors (NPYRs) belong to the G protein-coupled receptor family (Gehlert, 2004), at present, five receptor gene subtypes have been identified in mammals, namely *npy1r*, *npy2r*, *npy4r*, *npy5r,* and *npy6r* (Wraith et al., 2000). Based on pharmacological data of human and mouse cardiomyocytes, it is speculated that *npy3r* may exist, but it has not been cloned and characterized in any vertebrate, so it cannot exist as an independent gene (Lee and Miller, 1998; Larhammar et al., 2001; Pedragosa-Badia et al., 2013). In addition to the five known receptors, *npy7r* and *npy8r* were also identified in teleost fishes, while *npy7r* may have been lost in mammals (Larhammar and Salaneck, 2004; Sundström et al., 2013; Zhou et al., 2013; Wang et al., 2019). In particular, *npy8r* has two subtypes, *npy8ar* and *npy8br* respectively, which has been reported in zebrafish (Sundström et al., 2013), orange spotted grouper (Wang et al., 2014) and Chinese perch (Zhang et al., 2021).

According to amino acid sequence homology and functional characteristics, NPYRs can be divided into Y1 subfamily (NPY1R, NPY4R, NPY6R, and NPY8R), Y2 subfamily (NPY2R, NPY7R) and Y5 subfamily (NPY5R) (Larhammar and Salaneck, 2004). Different receptors regulate different functions, in *npy* receptor knockout mice, *npy1r* and *npy5r* knockout mice exhibited decreased food intake and body weight. *npy4r* knockout mice showed that *npy4r* promotes obesity induced by high fat diet (Wang et al., 2023). Deletion of *npy6r* resulted in body weight loss and late-life obesity in mice (Yulyaningsih et al., 2014). Injection of siRNA-*npy8br* into Chinese perch’s ventricle indicates that *npy8br* plays an important role in appetite regulation (Zhang et al., 2021).

At present, *npy2r* has been cloned and characterized in mammals including human (Larhammar et al., 1992; Gerald et al., 1995), mouse (Nakamura et al., 1996), pig (Wraith et al., 2000) and some teleost fishes such as rainbow trout (Larsson et al., 2006), orange-spotted grouper (Wang et al., 2014) and large yellow croaker (Wang et al., 2019). Immunohistochemical localization revealed that NPY2R is presynaptic receptor and is expressed in many regions of the amygdala in mouse (Stanić et al., 2011). In fish, it has been confirmed that zebrafish NPY2R is homologous to mammals and is most similar in pharmacology to chicken (Wang et al., 2019). Studies have shown that *npy2r* knockout mice gain weight, increase food intake, and increase fat deposition (Naveilhan et al., 1999). Through maze experiment, open field and light/dark test, it has been demonstrated that *npy2r* plays an important role in anxiety and stress-related behavior in mice (Tschenett et al., 2003). It has been shown that loss of *npy2r* in the hippocampus of mice decreases episodic fear memory, but improves working memory and spatial memory (Tschenett et al., 2003; Hörmer et al., 2018). Studies have shown that *npy2r* is highly expressed in the brain, liver and gonads of fish (Larsson et al., 2006; Wang et al., 2014; Wang et al., 2019). However, functional studies of *npy2r* are mainly focused on mammals such as human and mice, and only a few teleost fish have been preliminarily explored based on pharmacological characteristics, while the physiological function of *npy2r* in fish has not been investigated.

In order to explore the physiological function of *npy2r* in fish, in particular, whether fish *npy2r* also plays an important role in anxious behavior and feeding activity, it is necessary to establish gene knockout models. Japanese medaka (*Oryzias latipes*) is an important model organism, it has the advantages of transparent embryo, fast growth rate and easy cultivation. In this study, we used the CRISPR/Cas9 technology to knockout the *npy2r* gene in Japanese medaka, in order to further explore the physiological function of *npy2r* gene.

## 2 Materials and methods

### 2.1 Medaka maintenance

The male and female wild-type (WT) Japanese medaka HdrR (orange-red strain) were obtained from the Institute of Hydrobiology, Chinese Academy of Sciences (Wuhan, China). They were bred in the circulating water system of the Research Center of Mandarin Fish in Huazhong Agricultural University with 27°C of room temperature and 14 h of light and 10 h of darkness. Embryos were collected in the morning and incubated at 28°C incubator. The male WT, male *npy2r* ^*+/−*^ medaka and female *npy2r* ^+/+^ medaka used in this study were 5 months old.

### 2.2 *npy2r* sequence analysis

NCBI (https://www.ncbi.nlm.nih.gov/) and Ensembl (https://asia.ensembl.org/index.html) were used to search the NPY receptors genes and amino acid sequences of Japanese medaka, Human (*Homo sapiens*), Mouse (*Mus musculus*), Seabass (*Dicentrarchus labrax*), Swamp eel (*Monopterus albus*), Spotted gar (*Lepisosteus oculatus*), Nile tilapia (*Oreochromis niloticus*), Zebrafish (*Danio rerio*), Rainbow trout (*Oncorhynchus mykiss*), Atlantic cod (*Gadus morhua*), Chinese perch (*Siniperca chuatsi*), Japanese flounder (*Paralichthys olivaceus*), African clawed frog (*Xenopus laevis*), and Torafugu (*Takifugu rubripes*). The accession numbers are shown in Supplementary Table S1. Multiple amino acid alignment of *npy2r* among medaka and other Vertebrate were performed by using Clustal X, and the protein genealogies of NPY receptors were assessed by the Neighbor-Joining using Mega11.

### 2.3 Establishment of *npy2r* mutant line medaka by CRISPR/Cas9

The CCTOP web (https://cctop.cos.uni-heidelberg.de/) was used to design medaka single guide RNA (sgRNA). Two targets on the second exon of medaka *npy2r* gene were designed. The sgRNAs were then cloned into PMD-19T vector and synthesized using TranscriptAid T7 High Yield Transcription kit (Thermo, Scientific). Medaka embryos at the single-cell stage were microinjected with 2 nL of a mixed solution consisting of 1 μL of sgRNAs (50 ng/μL), 1 μL of Cas9 mRNA (300 ng/μL), 0.5 μL of phenol red indicator, and 2.5 μL of DEPC water. After 24 h of microinjection, genomic DNA was extracted from medaka embryos, and the effectiveness of sgRNAs were tested by PCR and sequencing. The microinjected embryos were cultured to adulthood. The caudal fin genome DNA was extracted, and the F0 mutant line was obtained by PCR amplification and agarose gel electrophoresis. After that, the F0 mutant (male) and WT (female) medaka were crossed to generate F1 heterozygous mutants. The F2 homozygous mutant medaka was obtained by crossing F1 of the same mutant type. The sgRNAs and detection primers of *npy2r* are shown in Table 1.

**TABLE 1:** sgRNA and detection primers of *npy2r* gene.

Primer	Sequences (5′-3′)	Tm (°C)
sgRNA1	GAT​GAC​AGT​ACA​AAA​CTG​GT	59
sgRNA2	TCT​ACA​CAC​TCT​ATG​ATG​AG	
*npy2r*-F	ACT​GAG​TGT​GCA​CAA​TGC​TTT​T	
*npy2r*-R	TGT​GCA​CAC​CTG​AAT​GGA​CT	59

### 2.4 Genotypic sex identification

In order to identify the genotypic sex of adult medaka, we used DNA extraction kit to extract DNA from the tail fin of medaka according to the manufacturer’s instructions. Sex-specific primers *dmrt1bY* (5′-AGA​GGA​GGA​GCT​TGG​GAT​TTG​TAG-3′) and *dmrt1a* (5′-CAG​ACG​CTT​CCT​CGC​CGT​AA-3′) were then used for PCR amplification (Patil and Hinze et al., 2008).

### 2.5 Gonad histological analysis

The male WT and male *npy2r* deficient male medaka were anesthetized with MS222, placed on ice and dissected immediately, and then the removed gonads were placed in fixative for 24 h. The gonads were dehydrated and embedded in paraffin. Paraffin sections were made using a sectioning mechanism and stained with hematoxylin and eosin (H&E).

### 2.6 Food intake measurement

The male WT, female *npy2r* ^+/+^ and male *npy2r* ^*+/−*^ medaka were randomly selected and placed under the same experimental conditions to determine the food intake. Before the experiment, the medaka were starved for 24 h. Before feeding, the medaka were weighed and placed individually in 1-L tank. The medaka were fed an excessive amount of brine shrimp (*Artemia nauplii*) and allowed to eat freely for 1 h, then the weight of medaka were measured again (Shi et al., 2020). The difference before and after feeding is the food intake of medaka.

### 2.7 Behavior analysis

#### 2.7.1 Mirror test

The mirror test, or mirror approaching behavior, is a good model for measuring social interaction and social anxiety (Ansai et al., 2016; Lucon-Xiccato et al., 2022). Mirror test was performed with reference to the method already reported (Shi et al., 2020; Shiozaki et al., 2020). In this experiment, a non-transparent rectangular tank (20 × 10 × 10 cm) filled with water was used, and put a mirror on one side. The male WT, female *npy2r* ^*+/+*^ and male *npy2r* ^*+/−*^ medaka were randomly selected, one fish at a time, and slowly placed into the tank for 2 min to adapt to the environment. After that, they were allowed to explore freely without being disturbed (*n* = 7). The movement trajectory for 10 min was recorded, EthoVision XT software was used to analyze the biting time and swimming distance.

#### 2.7.2 Open-field test

The Open-field test can be used to assess medaka anxiety levels and locomotor activity of medaka (Lucon-Xiccato et al., 2022). In this experiment, a non-transparent rectangular tank (20 × 10 × 10 cm) filled with water was used, and a camera was placed above the tank to record the movement trajectory of medaka. The male WT, female *npy2r* ^*+/+*^ and male *npy2r* ^*+/−*^ medaka were randomly selected, one fish at a time, and slowly placed into the tank for 2 min to adapt to the environment. After that, they were allowed to explore freely without being disturbed (*n* = 7). The movement trajectory for 10 min was recorded, and EthoVision XT software was used to analyze the swimming distance, movement time and total freezing time.

### 2.8 qRT-PCR analysis

The expression levels of anxiety and appetite genes were analyzed by real-time quantitative PCR (qRT-PCR) using medaka brain cDNA as template. The eye, brain, gill, heart, kidney, ovary, testis, liver and spleen from medaka were sampled for tissue expression analysis of *npy2r*. The total RNA was extracted by TRIzol Reagent (Takara, Japan), and then 1 μg RNA was reverse transcribed into cDNA using reverse transcription kit (Vazyme, China). SYBR (Vazyme, China) and *th1* (tryptophan hydroxylase1), *th2*, *agrp* (agouti-related protein), *cck* (cholecystokinin), pomc (pro-opiomelanocortin), *npy* (neuropeptide Y), *gr1* (glucocorticoid receptor 1), *gr2*, *mr* (mineralocorticoid receptor) genes specific primers were used for qRT-PCR. Table 2 shows the specific primers sequences of *npy2r*, *th1*, *th2*, *agrp*, *cck*, *pomc*, *npy*, *gr1*, *gr2,* and *mr* genes. The 20 μL RT-qPCR reaction system included 1 μL cDNA, 0.5 μL forward and reserve primers (10 mmol/μL), 10 μL SYBR, and 8 μL double distilled water (ddH2O). The conditions for PCR were as follows: 95°C for 3 min initially, followed by 40 cycles at 95°C for 10 s, 58°C for 30 s and 72°C for 30 s, and melting curve assay from 65°C gradually increasing 0.5°C s−1°C to 95°C, with acquisition data at every 6 s. The mRNA expression levels of target genes were quantified relative to the expression of *β-actin* using the optimized comparative Ct (2^−ΔΔCt^) value method (Livak and Schmittgen, 2001).

**TABLE 2:** Primer sequences for the quantitative real-time PCR.

Primer	Sequences (5′-3′)
*β-actin*-F	TTTATGCCAGCAACGACT
*β-actin*-R	CGA​CGA​AAG​CCC​TAC​TCC​C
*npy2r*-RT-F	CGT​GCA​CCA​CAT​GGA​GAC​TA
*npy2r*-RT-R	CCA​CTT​CTC​TGT​GCA​CAC​CT
*th1*-F	TCC GTT CACCCA CAA CAT AG
*th1*-R	AAC CTT CAG CTC GTC CTT CA
*th2*-F	GTT GAG GAA CAC GTC CAG GT
*th2*-R	CGT TCG AAG CCA AAC TTC TC
*agrp*-F	GCA​TCC​CTC​ACC​AGC​AGT​C
*agrp*-R	GCC​TAT​TTG​GCG​GCA​GTA​AC
*cck*-F	TCC​TTC​TGA​AGT​TGC​TCT​T
*cck*-R	CCGTGAATCTCCATCCTC
*pomc*-F	TTG​CTG​GCT​GTT​GGT​GGT​TCT
*pomc*-F	AGG​TCT​GGG​CTT​TCA​GGT​TTG​A
*npy*-F	GCC​TTG​GAG​CCT​TAA​CAG​AGG
*npy*-R	TCT​CAG​GAC​TGG​ACC​TCT​TCC
*gr1*-F	GCG​AGA​TAA​GAC​CCG​AAG​CA
*gr1*-R	GCC​TTT​AGT​TCC​ACC​TTG​TCC​A
*gr2*-F	GAG​CAG​GAC​CCC​ATT​GAT​CTT
*gr2*-R	AGC​ATC​GTG​CCC​AAC​GTA​AA
*mr*-F	CCA​GAG​GTG​AAG​GGT​ATC​CA
*mr*-R	GAA​GCC​TCG​TCT​CCA​CAA​AC

Abbreviations: *β-actin*, beta-actin; *th1*, tryptophan hydroxylase1; *agrp*, agouti-relate protein; *cck,* cholecystokinin; *pomc*, pro–opiomelanocortin; *npy*, neuropeptide Y; *gr1*, glucocorticoid receptor 1; *mr*, mineralocorticoid receptor.

### 2.9 Statistical analysis

The SPSS 25.0 software was used for statistical analysis. All data were expressed as means ± S.E.M, and analyzed by independent-samples *t*-test. GraphPad Prism 8.0.2 used to make data analysis diagram, *p* < 0.05 and *p* < 0.01 were considered statistically significant.

## 3 Results

### 3.1 Sequence analysis of medaka *npy2r*

**FIGURE 1:**
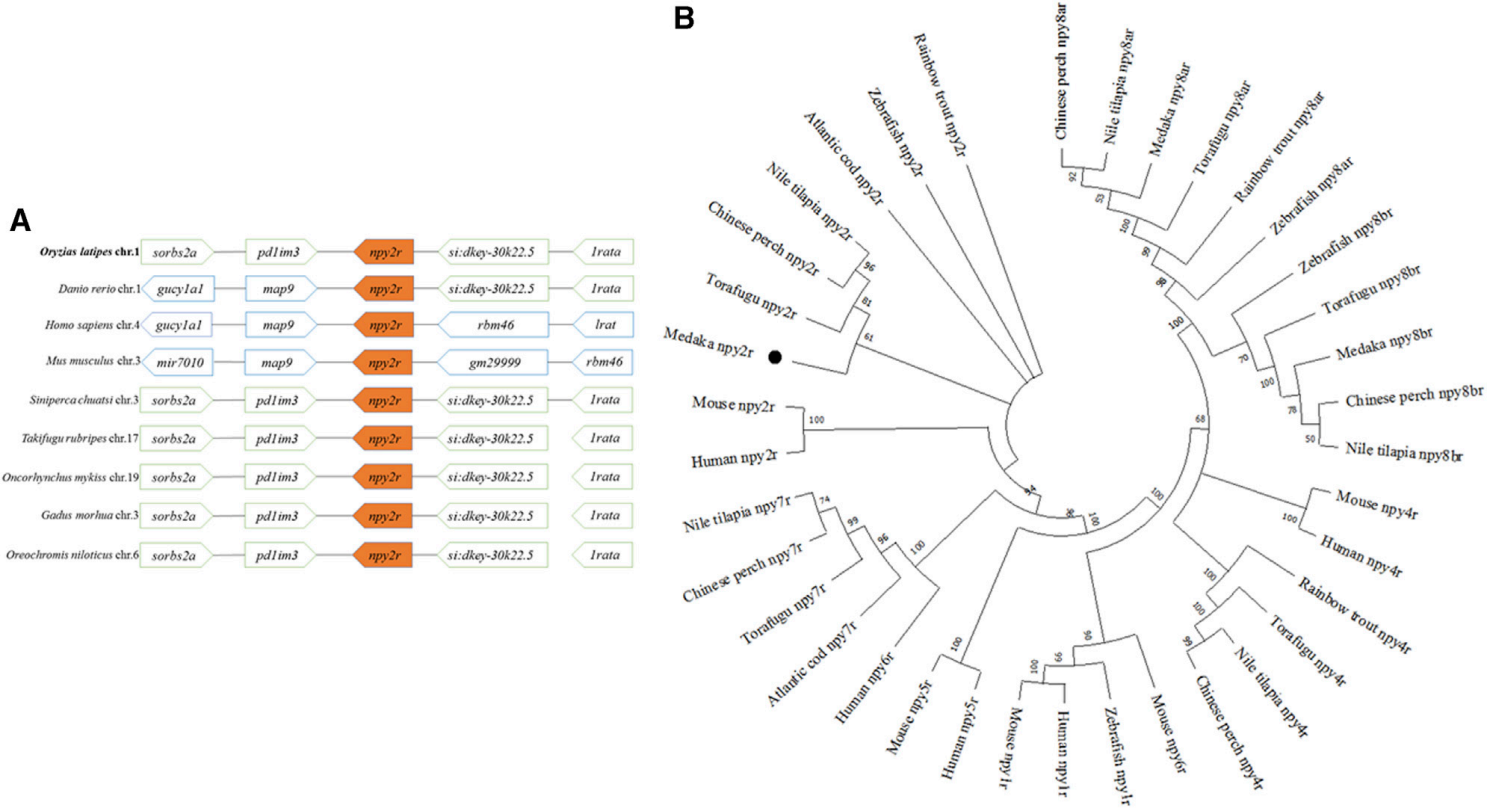
Amino Acid Sequence analysis of *npy2r*. **(A)**: Synteny analyses of *npy2r* in medaka, zebrafish, human, mouse, Chinese perch, Torafugu, Rainbow trout, Atlantic cod and Nile tilapia. **(B)**: Phylogenetic analysis of *npy2r* in vertebrates.

Amino acid sequence alignment in vertebrates revealed low conservation of *npy2r*. The *npy2r* amino acid sequence of medaka showed 79.2% homology with seabass, 74.9% homology with swamp eel, 55.7% homology with human and 49.2% homology with mouse, respectively (Supplementary Figure S1). Comparing the medaka, *npy2r* adjacent genes with other vertebrates, synteny analysis showed that the *npy2r* adjacent genes were highly conserved among the medaka, Chinese perch, Torafugu, Atlantic cod, Rainbow trout and Nile tilapia, but showed different synteny from human and mouse (Figure 1A). The phylogenetic tree showed that medaka *npy2r* is homologous to human and mouse *npy2r*, moreover, medaka is more closely related to Torafugu, Chinese perch and Nile tilapia (Figure 1B).

### 3.2 Establishment of *npy2r* mutant line medaka

**FIGURE 2:**
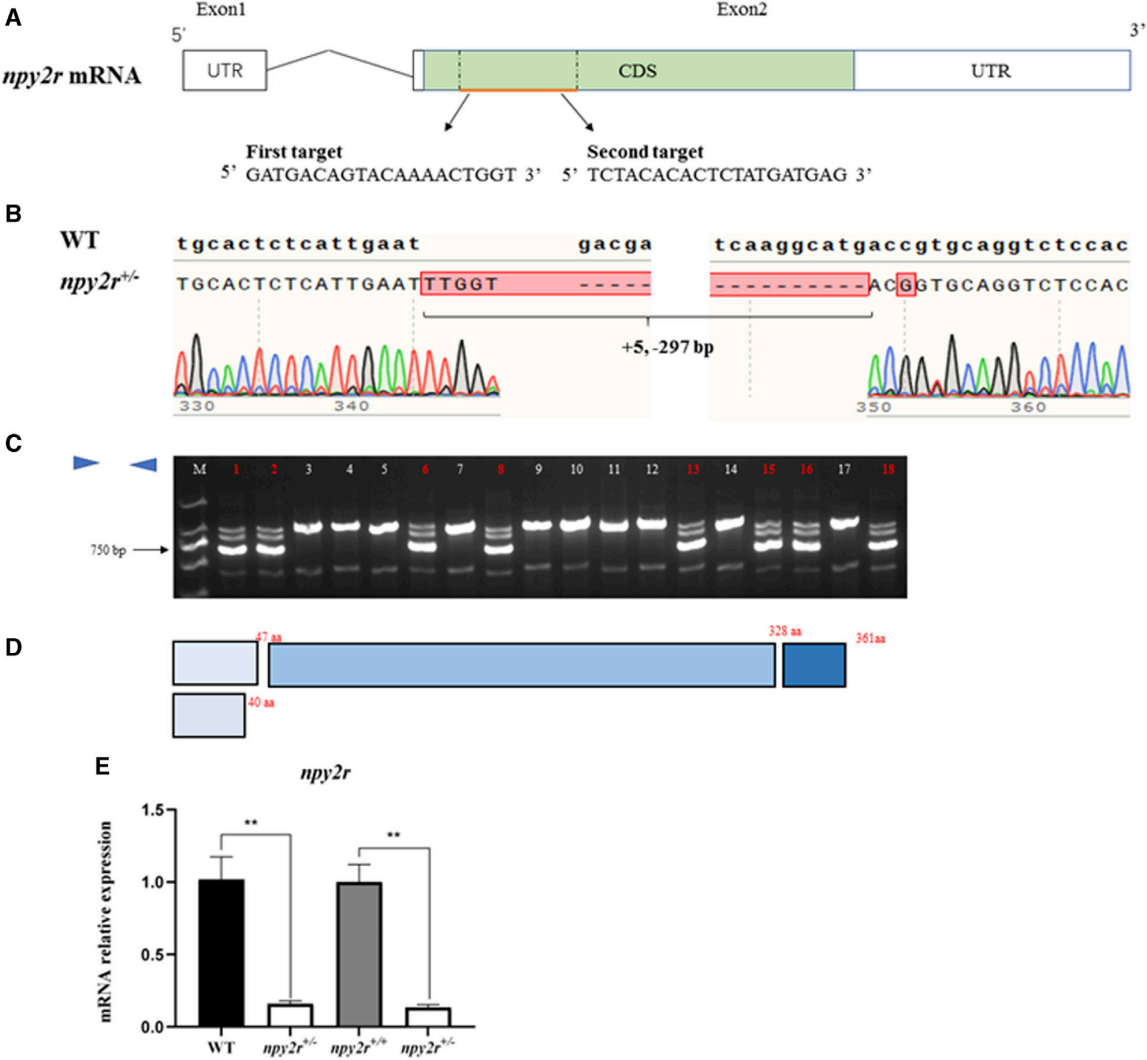
Establishment of *npy2r* mutant line medaka. **(A)**: The *npy2r* gene structure was composed of two exons (white box) and an intron (black line), in which the mRNA coding sequence (CDS) (green box) is entirely present in exon2. The genomic target of CRISPR/Cas9 is located in the CDS region. **(B)**: The result of WT and *npy2r* ^*+/−*^ DNA sequencing showed that the mutant had 297 bp deletion, 5 bp addition and 1bp mutation. **(C)**: Agarose gel electrophoresis. M:marker; The lanes marked by red numbers are *npy2r* ^*+/−*^ and the white numbers are *npy2r* ^*+/+*^. **(D)**: Comparison of *npy2r* ^*+/−*^ mutant with *npy2r* ^*+/+*^ protein structure. **(E)**: mRNA expression level of *npy2r* ^*+/−*^ medaka. * represents significant differences exist between WT and *npy2r* ^*+/−*^ medaka or *npy2r* ^*+/+*^ and *npy2r* ^*+/−*^ medaka. Data were presented as means ± S.E.M. (*n* = 6). * *p* < 0.05, * * *p* < 0.01.

The genome target was located in the second exon, and the F0 mutant was obtained by microinjection (Figure 2A). The F1 heterozygote (*npy2r* ^*+/−*^) was obtained by PCR and sequencing after hybridization of FO and WT medaka, namely 297 bp deletion, 5 bp addition and 1 bp mutation (Figure 2B). Agarose gel electrophoresis showed that *npy2r* ^*+/+*^ had only one bright band at approximately 1,000 bp, while *npy2r* ^*+/−*^ had three bands (Figure 2C). The additional insertion, deletion, and mutation of the bases resulted in premature termination of *npy2r* gene translation compared with WT. As a result, the protein only had 44 amino acids and the conserved 7 transmembrane domains of the *npy2r* protein were completely lost (Figure 2D). The mRNA level of *npy2r* was significantly decreased in *npy2r* ^*+/−*^ medaka (Figure 2E).

### 3.3 *npy2r* deficient medaka had sex development disorder

**FIGURE 3:**
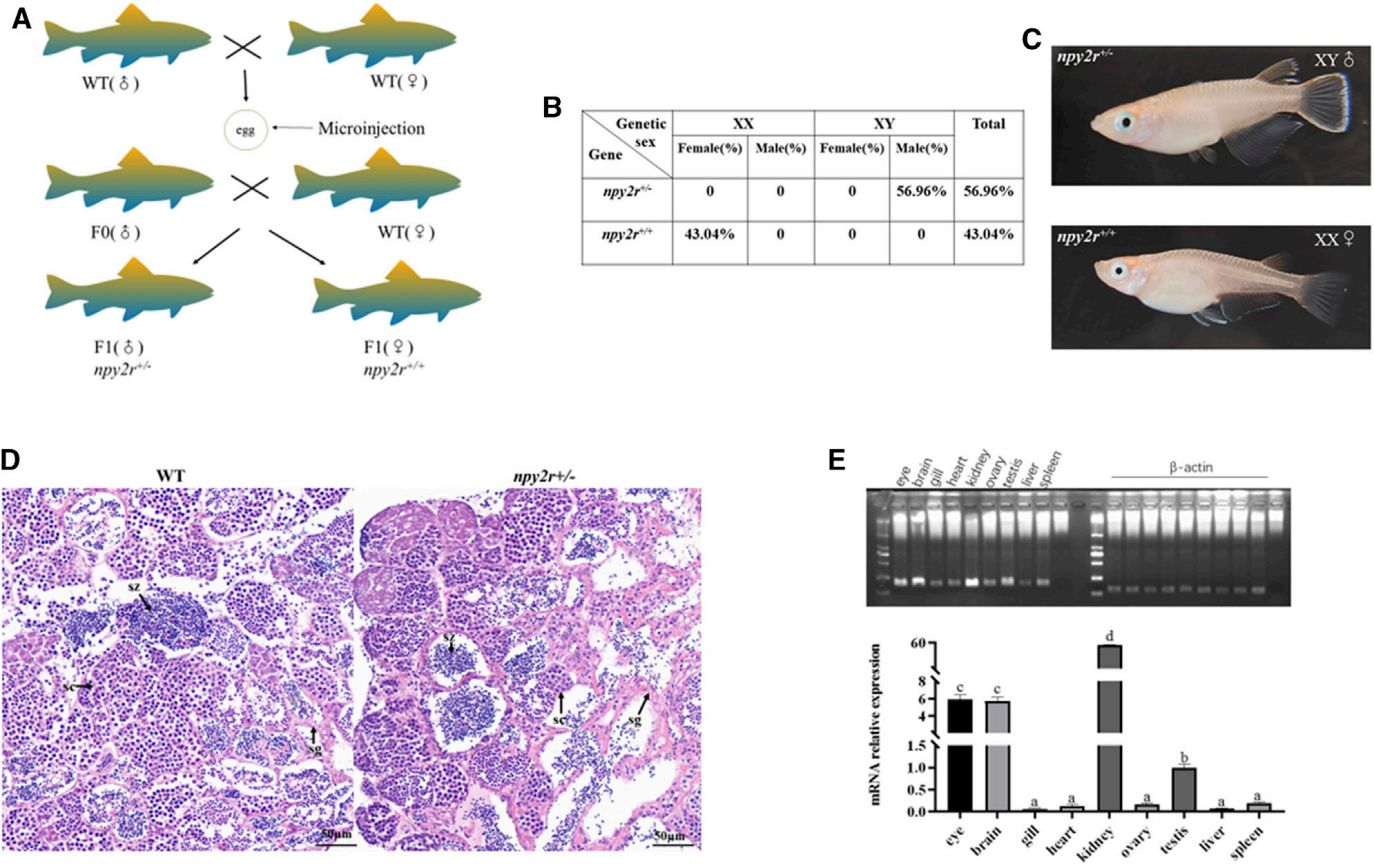
*npy2r* mutant line medaka had sex development disorder. **(A)** Genotypes and sex types of medaka. **(B)** The proportion of genetic sex in the F1 generation of medaka, *n* = 200. **(C)** Sexual phenotype of F1 generation of medaka. **(D)**: Gonad histology of WT and *npy2r* ^*+/−*^ medaka. Scale bar: 50 μm. sg, spermatogonia; sc, spermatocytes; sz, spermatozoa. **(E)**: *npy2r* tissue expression differences were analyzed by polymerase chain reaction (PCR) and qRT-PCR. Data were presented as means ± S.E.M. (*n* = 6). Different small letters above the bars indicate significant differences at *p* < 0.05.

In the process of creating homozygous mutant, based on the shape of the dorsal fin and anal fin, it was found that the *npy2r* ^*+/−*^ medaka in F1 generation were exclusively male, while the *npy2r* ^*+/+*^ medaka were exclusively female. Therefore, we conducted genetic sex identification to determine whether there was sex reversal in the F1 mutant. The results showed that the genetic sex of F1 medaka were consistent with the physiological sex, that is, there was no sex reversal (Figures 3A, C). Our statistics showed that *npy2r* ^*+/−*^ medaka accounted for 56.96% and *npy2r* ^*+/+*^ medaka accounted for 43.04% in the F1 medaka (Figure 3B). H&E sections of the gonad revealed that spermatogonia, spermatogonia, spermatocytes and spermatozoa developed normally in WT and *npy2r* ^*+/−*^ medaka (Figure 3D). Tissue expression analysis showed that *npy2r* was expressed in the gonad of medaka (Figure 3E).

### 3.4 Food intake and growth performance of *npy2r* deficient medaka

**FIGURE 4:**
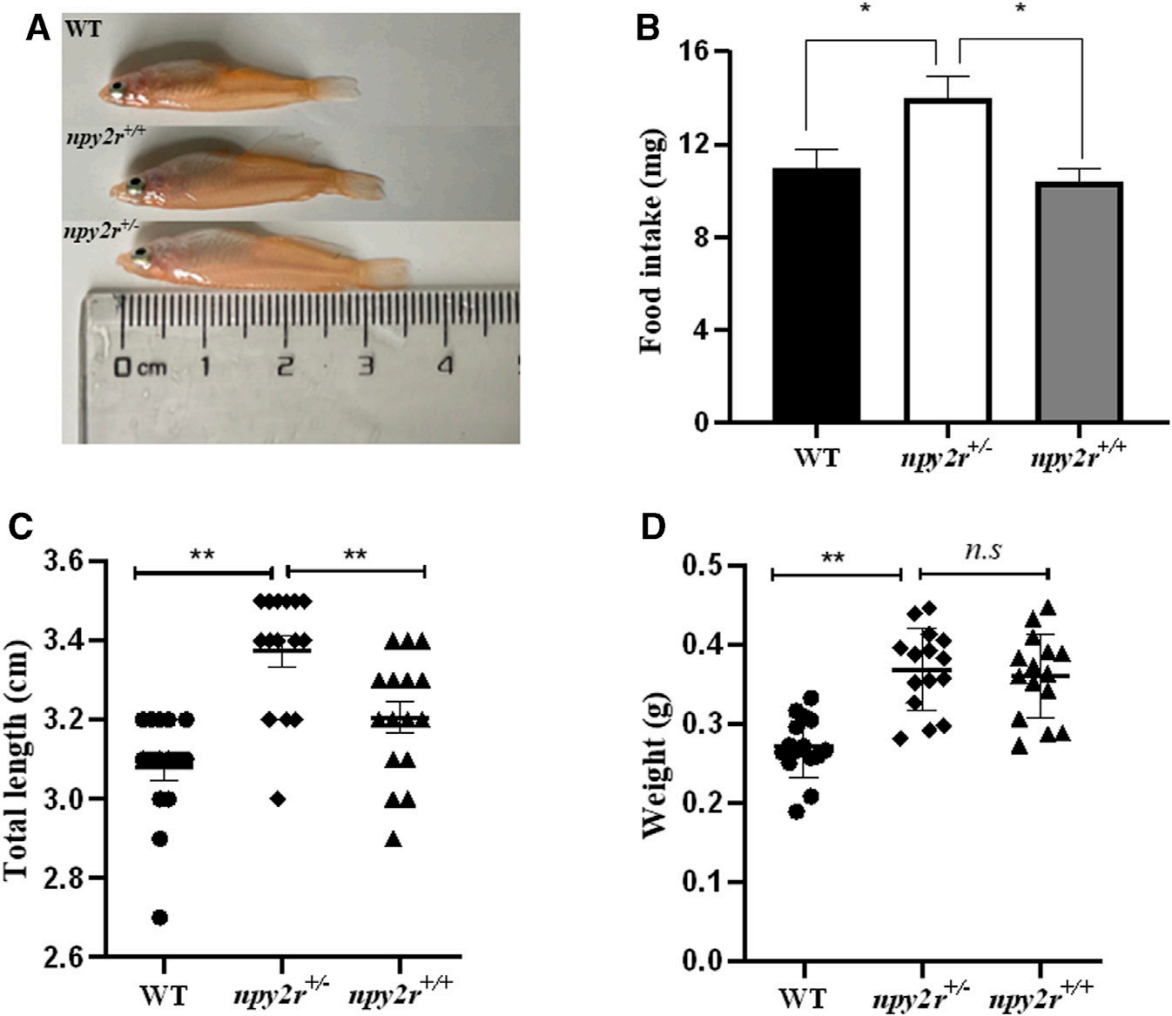
Growth performance of *npy2r* deficient medaka. **(A)**:Appearance of WT, *npy2r* ^*+/+*^ and *npy2r* ^*+/−*^ medaka. **(B)**: Food intake of WT, *npy2r* ^*+/+*^ and *npy2r* ^*+/−*^ medaka (WT, n = 9; *npy2r* ^*+/−*^, n = 10; *npy2r* ^*+/+*^, *n* = 10). **(C)**: Total length of WT, *npy2r* ^*+/+*^ and *npy2r* ^*+/−*^ medaka. **(D)**: Body weight of WT, *npy2r* ^*+/+*^ and *npy2r* ^*+/−*^ medaka. (*n* = 15). Data were presented as means ± S.E.M. * *p* < 0.05, * * *p* < 0.01, *n.s*, not significant.

The medaka was fed freely for 1 h, the food intake statistics showed that the food intake of *npy2r* ^*+/−*^ medaka was higher than WT and *npy2r* ^*+/+*^ medaka, and there were significantly different between *npy2r* ^*+/−*^ medaka and both WT and *npy2r* ^*+/+*^ medaka (*p <* 0.05) (Figure 4B). By measuring the growth performance of WT, *npy2r* ^*+/+*^ and *npy2r* ^*+/−*^ medaka, we found that the total length and body weight of *npy2r* ^*+/−*^ medaka increased significantly (*p <* 0.01) (Figures 4A, C, D). Compared with *npy2r* ^*+/+*^ medaka, the total length of *npy2r* ^*+/−*^ medaka was significantly increased (*p <* 0.01), but the body weight was not significantly different between *npy2r* ^*+/+*^ and *npy2r* ^*+/−*^ medaka (*p >* 0.05) (Figures 4C, D).

### 3.5 Mirror test analysis of *npy2r* deficient medaka

**FIGURE 5:**
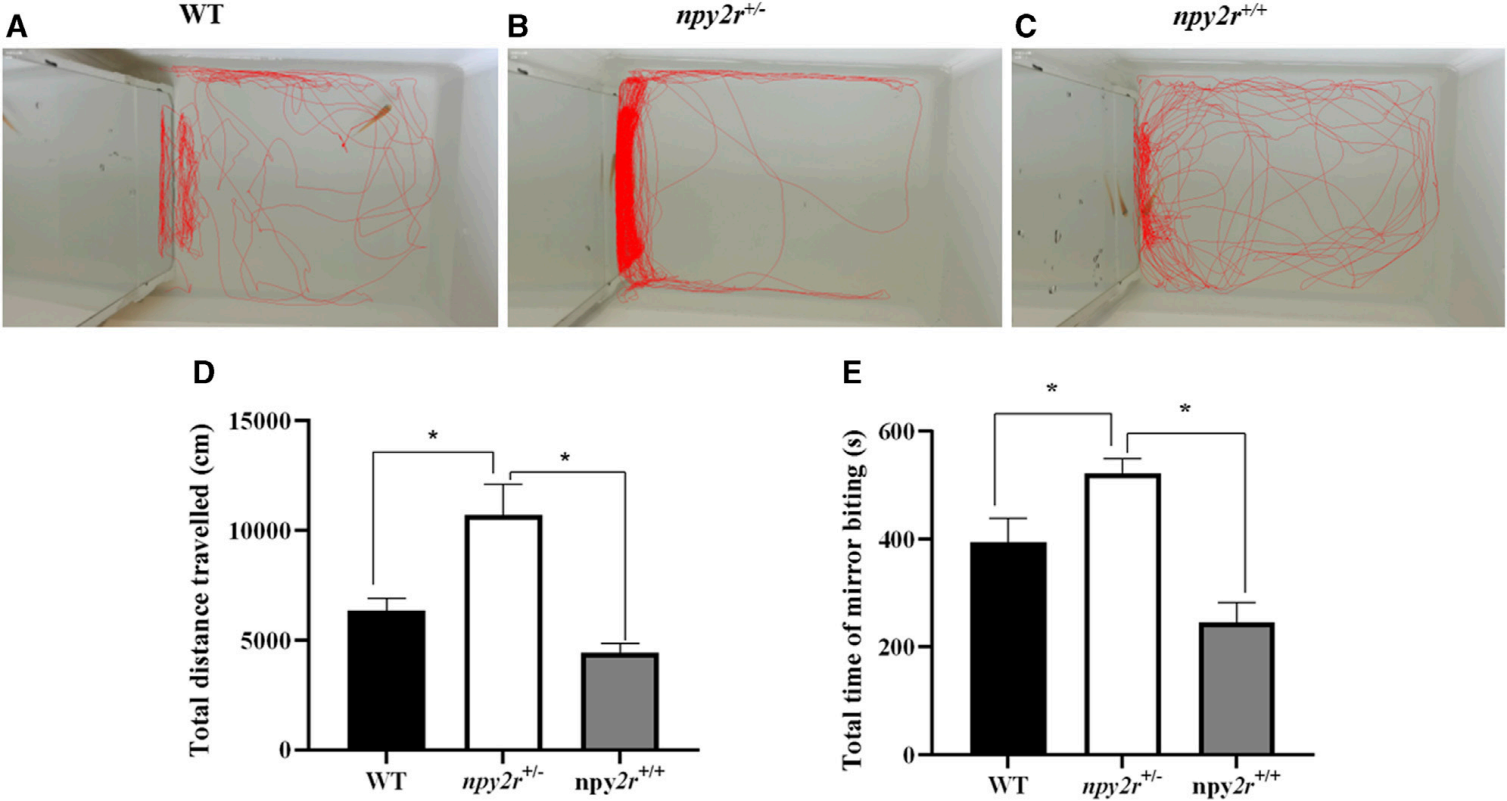
Mirror test analysis of *npy2r* deficient medaka. **(A)**: WT medaka swimming trajectories. **(B)**: *npy2r* ^*+/+*^ medaka swimming trajectories. **(C)**: *npy2r* ^*+/−*^ medaka swimming trajectories. **(D)**: Total distance travelled. **(E)**: Total time of mirror biting. Data were presented as means ± S.E.M. (*n* = 7). * represents significant differences exist between WT and *npy2r* ^*+/−*^ medaka or *npy2r* ^*+/+*^ and *npy2r* ^*+/−*^ medaka. *p* < 0.05.

In the mirror test, the medaka will infer the presence of another fish based on the mirror and then display either touching or biting the mirror. Therefore, the social behavior of medaka can be evaluated by the mirror test. In this study, according to the WT, *npy2r* ^*+/+*^ and *npy2r* ^*+/−*^ medaka three-dimensional trajectory graph can clearly see *npy2r* ^*+/−*^ medaka swam back and forth in one side of the mirror, however, WT and *npy2r* ^*+/+*^ medaka swam all over the region (Figures 5A–C). Compared with WT and *npy2r* ^*+/+*^ medaka, *npy2r* ^*+/−*^ medaka significantly increased the total distance travelled and contact time with the mirror during the whole behavior test (*p <* 0.05) (Figures 5D, E).

### 3.6 Open field test analysis of *npy2r* deficient medaka

**FIGURE 6:**
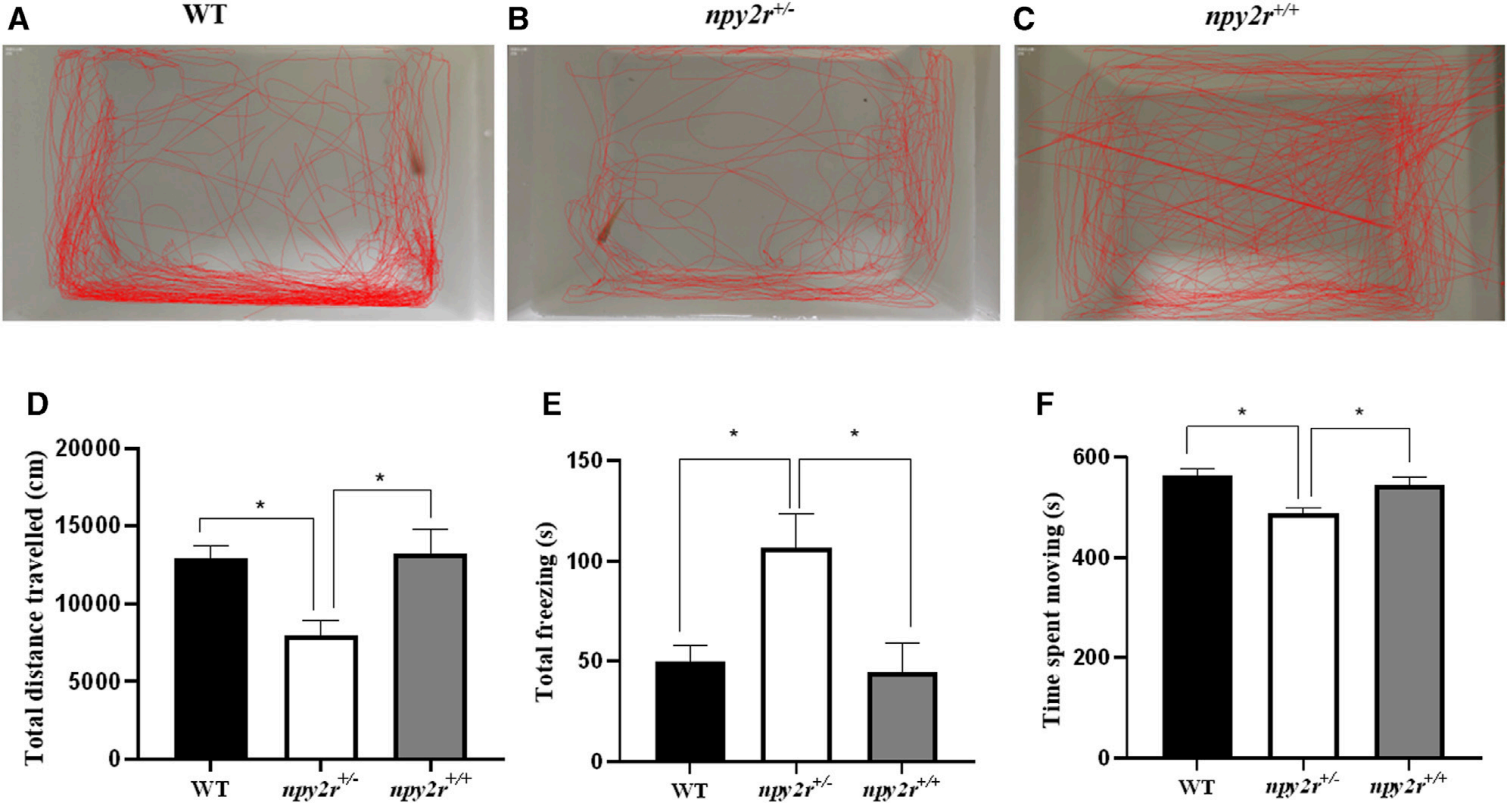
Open-field test behavior analysis of *npy2r* deficient medaka. **(A)**: WT medaka swimming trajectories. **(B)**: *npy2r* ^*+/−*^ medaka swimming trajectories. **(C)**: *npy2r* ^*+/+*^medaka swimming trajectories. **(D)**: Total distance travelled. **(E)**: Total freezing. **(F)**: Time spent moving. Data were presented as means ± S.E.M. (*n* = 7). * represents significant differences exist between WT and *npy2r* ^*+/−*^ medaka or *npy2r* ^*+/+*^ and *npy2r* ^*+/−*^ medaka. *p* < 0.05.

When placed in a new environment, the medaka display exploratory behavior in both marginal and central areas, thus, it is possible to assess the anxiety of medaka by their locomotor behavior. In open field test, the movement trajectories of WT, *npy2r* ^*+/+*^ and *npy2r* ^*+/−*^ medaka are shown in Figure 4. WT and *npy2r* ^*+/−*^ medaka are more inclined to explore the edges (Figures 6A–C). Compared with WT and *npy2r* ^*+/+*^ medaka, the total distance travelled and movement time of *npy2r* ^*+/−*^ medaka decreased significantly (*p <* 0.05) (Figures 6D, E), while the total freezing time was significantly increased (*p <* 0.05) (Figure 6F).

### 3.7 Gene expression level of *npy2r* deficient medaka

**FIGURE 7:**
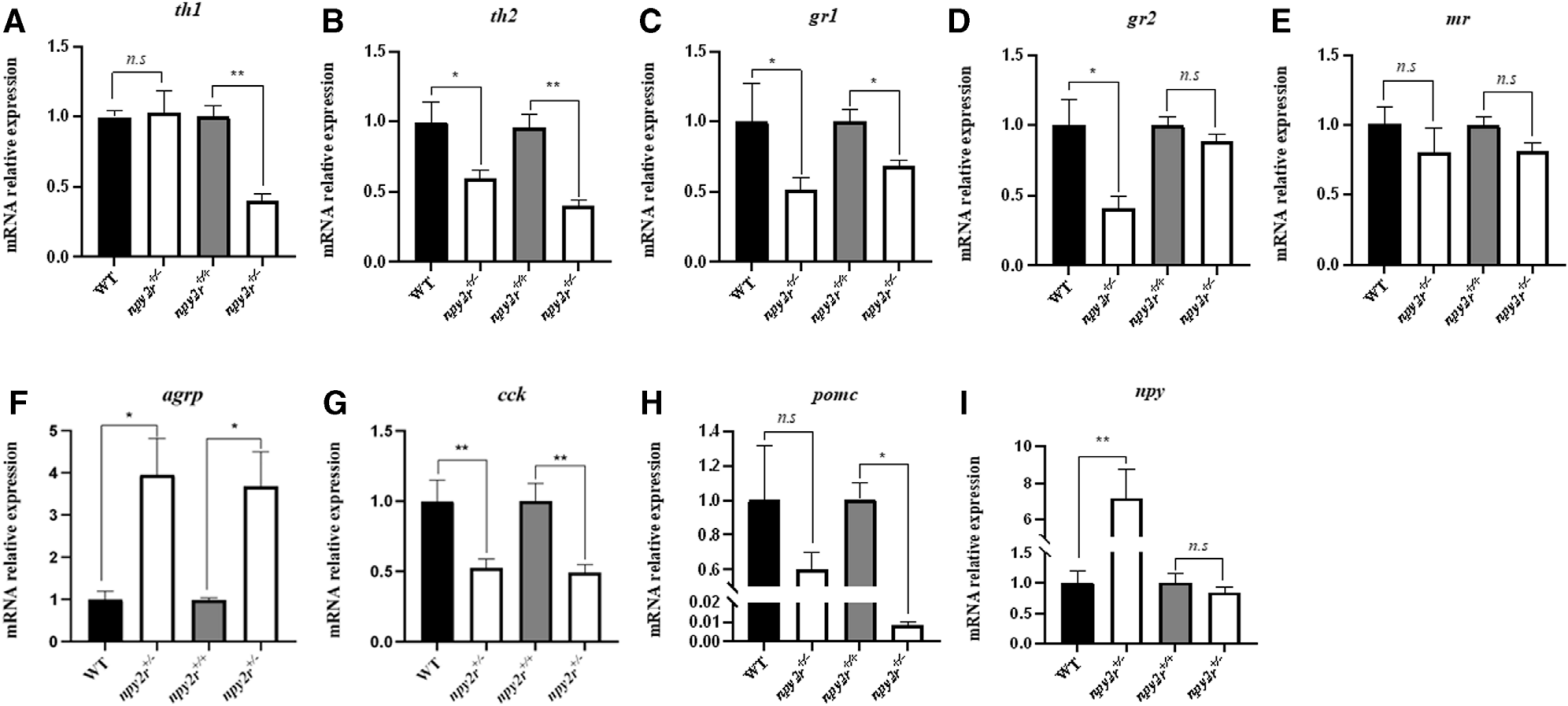
The mRNA levels of *th1* **(A)**, *th2* **(B)**, *gr1* **(C)**, *gr2* **(D)**, *mr* **(E)**, *agrp* **(F)**, *cck* **(G)**, *pomc* **(H)**, *npy* **(I)** in WT, *npy2r* ^*+/+*^ and *npy2r* ^*+/−*^ medaka. The mRNA levels in *npy2r* ^*+/−*^ medaka is shown as a relative value to that in WT or *npy2r* ^*+/+*^ medaka in each gene analysis. Data were presented as means ± S.E.M. (*n* = 6). *p < 0.05, **p < 0.01, *n.s*, not significant.

In order to compare the expression patterns of anxiety and appetite related genes of WT, *npy2r* ^*+/+*^ and *npy2r* ^*+/−*^ medaka, we measured the mRNA expression levels of genes associated with anxiety behavior (*th1*, *th2, gr1*, *gr2*, *mr*), appetitive genes (*agrp*, *npy*), and anorexigenic factors (*pomc*, *cck*) by qRT-PCR. The results showed that compared with WT, the expression levels of *th2*, *gr1*, *gr2*, *cck* gene in *npy2r* ^*+/−*^ medaka were significantly decreased (*p <* 0.05) (Figures 7B–D, G), *agrp*, *npy* expression levels of *npy2r* ^*+/−*^ medaka were significantly increased (*p <* 0.05) (Figures 7F, I), and *th1*, *mr*, and *pomc* were not significantly different between WT and *npy2r* ^*+/−*^ medaka (*p >* 0.05) (Figures 7A, E, H). Compared with *npy2r* ^*+/+*^, the expression levels of *th1*, *th2*, *gr1*, *cck* and *pomc* in *npy2r* ^*+/−*^ medaka were significantly decreased (*p <* 0.05) (Figures 7A–C, G, H), *agrp*, *npy* expression levels of *npy2r* ^*+/−*^ medaka were significantly increased (*p <* 0.05) (Figures 7F, I), and *gr2*, *mr* and *npy* were not significantly different between *npy2r* ^*+/+*^ and *npy2r* ^*+/*^ medaka (*p >* 0.05) (Figures 7D, E, I).

## 4 Discussion

NPY is composed of 36 highly conserved amino acids and forms the NPY family together with peptide YY and pancreatic peptides (Cerdá-Reverter et al., 2000; Hansel et al., 2001; Conlon, 2002). NPY and its receptors have been studied extensively in the past few decades. The NPY receptor family eventually produced seven receptor subtypes named NPY1R, *NPY2R*, NPY4R, NPY5R, NPY6R, NPY7R, and NPY8R through two whole-genome duplications in the early stage of vertebrate evolution (Larsson et al., 2009; Sundström et al., 2013; Xu et al., 2015). NPY receptors have different receptor gene deletions in mammals and teleost fish, in this study, the phylogenetic tree showed that medaka *npy2r* is homologous to human and mouse *npy2r*.

At present, the establishment of *npy2r* knockout mice model revealed that *npy2r* plays an important role in anxiety behavior, feeding activity and learning (Naveilhan et al., 1999). However, it is not clear whether *npy2r* has the similar function in fish as in mammals, especially the knowledge related to anxiety and feeding. Based on this, we established *npy2r* mutant medaka by CRISPR/Cas9 system, namely 297 bp deletion, 5 bp addition and 1 bp mutation. The additional insertion, deletion, and mutation of the bases resulted in premature termination of *npy2r* gene translation compared with WT. As a result, the protein only had 44 amino acids and the conserved 7 transmembrane domains of the *npy2r* protein were completely lost. Meanwhile, the mRNA level of *npy2r* was significantly reduced in *npy2r* ^*+/−*^ medaka. Unexpectedly, during the establishment of *npy2r* homozygous mutant medaka, we found that *npy2r* ^*+/−*^ medaka were all-male. Therefore, *npy2r* ^*+/−*^ medaka could not be produced by self-fertilization of *npy2r* ^*+/−*^ medaka. H&E sections of gonad showed that the spermatogonia, spermatocytes and spermatozoa of *npy2r* ^*+/−*^ medaka developed normally and had normal fertility. Tissue expression analysis showed that *npy2r* was highly expressed in the testis and ovary of medaka. Studies have shown that *npy2r* may be involved in ovarian metabolism based on the *npy2r* expression profile and ligand binding characteristics of orange-spotted grouper (Wang et al., 2014). Consistently, tissue expression analysis of *npy2r* in rainbow trout suggested that *npy2r* may play a role in reproductive regulation (Larsson et al., 2006). However, no relevant reports were reported in *npy2r* knockout mice models. Therefore, *npy2r* knockout may affect the ovarian metabolism in medaka, resulting in the *npy2r* ^*+/−*^ medaka being all-male.

We investigated the effect of *npy2r* on food intake and the result showed that the food intake of *npy2r* ^*+/−*^ medaka increased significantly. Moreover, the total length and body weight were significantly increased compared with WT. The growth pattern of fish is different from higher vertebrates, which continue to grow until they reach a critical size. Studies have shown that fish are one of the animals with the highest efficiency in converting food into body tissue (Talbot, 1993). Therefore, the increase in food intake of *npy2r* ^*+/−*^ medaka will ultimately be reflected in growth indicators. We detected the expression of appetite genes by qRT-PCR, and the results showed that the knockout of *npy2r* in medaka significantly increased the expression of appetite stimulating genes (*agrp*, *npy*), and significantly decreased the expression of anorexia factors (*cck*, *pomc*), which further indicated that *npy2r* play an important role in feeding regulation of medaka. Previous studies have shown that *npy2r* knockout mice body weight and food intake were significantly increased (Naveilhan et al., 1999), and our study is consistent with previous research. Based on the exploration of *npy2r* gene in orange-spotted grouper, it is speculated that *npy2r* may play an important role in metabolism and energy balance (Wang et al., 2014). Studies have shown that *npy2r* is widely expressed in the brain, stomach and intestines of large yellow croaker, so *npy2r* may be involved in food intake and body weight regulation (Wang et al., 2019). Therefore, our research shows that *npy2r* plays an important role in fish feeding regulation.

Elevated mazes, open fields, light/dark tests are commonly used to evaluate anxiety-like behaviors in rodents such as mice (Bourin et al., 2007; Hurst and West, 2010). Similarly, there are many behavioral tests in fish. Since most animals, such as birds and fish, do not recognize their own mirror images, the mirror test is often used to assess sociability (Cattelan et al., 2017). Studies have shown that the mirror test is a good model for measuring social interaction and social anxiety (Ansai et al., 2016; Lucon-Xiccato et al., 2022). The medaka uses the visual stimulus formed by the mirror response to approach the mirror or bite the mirror. Thus, the sociability of the species can be assessed by the number of times they bite the mirror or the amount of time they spend in the area near the mirror (Lucon-Xiccato et al., 2022). In the present study, as shown by mirror test, *npy2r* knockout resulted in medaka significantly increased the total distance travelled and contact time with the mirror during the whole behavior test. Therefore, *npy2r* may play an important role in the social preference and sociability of the species.

The open-field test can be used to reflect the anxiety level and locomotor activity in medaka (Lucon-Xiccato et al., 2022). Therefore, we used the open field to evaluate the anxious behavior of *npy2r* deficient medaka. The results showed that *npy2r* knockout caused the total distance travelled and movement time of *npy2r* ^*+/−*^ medaka decreased, while the total freezing time increased. Previous studies have demonstrated that *npy2r* plays an important role in anxiety and stress-related behavior in mice using elevated maze, open field, and light/dark tests (Tschenett et al., 2003), and our study is consistent with previous research. Studies have shown that the medaka exhibit an anxious response when placed in a new environment, that is, it may first explore the entire region before showing a preference for edges (Hong and Zha, 2019; Lucon-Xiccato et al., 2022). Medaka will attempt to escape open filed in anxious situations, resulting in vigorous swimming (Lucon-Xiccato et al., 2020). We detected the expression levels of anxiety-related genes (*th1*, *th2, gr1*, *gr2*, *mr*) by qRT-PCR, the results showed that the expression levels of *th1*, *th2, gr1*, *gr2*, and *mr* gene in *npy2r* ^*+/−*^ medaka decreased significantly. Since *th1* and *th2* catalyze the production of dopamine, excessive exercise may lead to significantly increased expression of *th1* and *th2* genes in the brain (Otsuka et al., 2022). Studies have shown that the expression levels of *th1*, *th2*, *gr* and *mr* gene are significantly increased when zebrafish are in a state of anxiety or stress (Shiozaki et al., 2020). Therefore, the present study suggests that *npy2r* is involved in the regulation of anxiety behavior in medaka.

In conclusion, in this study, we established the *npy2r-*deficient medaka. It was found that the deletion of *npy2r* resulted in the *npy2r* ^*+/−*^ medaka were all-male, therefore, *npy2r* homozygous mutant lines could not be established. The study of *npy2r* ^*+/−*^ medaka showed that the absence of *npy2r* can improve social interaction and reduce anxiety behavior. In addition, the deletion of *npy2r* gene can promote the feeding and significantly increase the total length and weight in medaka. So far, this is the first *npy2r* gene knockout model established in fish and demonstrates that *npy2r* plays an important role in the regulation of reproduction, feeding and anxiety in fish.

## Data Availability

The original contributions presented in the study are included in the article/Supplementary material. Further inquiries can be directed to the corresponding author.
