# Supplementary material for: Neuropeptide Y receptor Y2 (npy2r) deficiency reduces anxiety and increases food intake in Japanese medaka (Oryzias latipes)
Source: Front Cell Dev Biol. 2023 Nov 7;11:1273006. doi: 10.3389/fcell.2023.1273006 (PMC10662287; doi:10.3389/fcell.2023.1273006)
Supplement: Supplementary file 1 [file Table1.DOCX]

Supplementary Material

# Supplementary Figures and Tables

## Supplementary Table1

**Supplementary Table 1. Accession numbers of NPY receptors**

| Gene | Species | Accession numbers |
| --- | --- | --- |
| *npy1r* | Human  Mouse  Zebrafish | [XP_005263088.1](https://www.ncbi.nlm.nih.gov/protein/XP_005263088.1)  [NP_001345884.1](https://www.ncbi.nlm.nih.gov/protein/NP_001345884.1)  [NP_001095861.1](https://www.ncbi.nlm.nih.gov/protein/NP_001095861.1) |
| *npy2r* | Human  Mouse  Medaka  Zebrafish  Nile tilapia  Rainbow trout  Atlantic cod  Chinese perch  Torafugu  Seabass  Swamp eel  Spotted gar  Japanese flounder  African clawed frog | [NP_000901.1](https://www.ncbi.nlm.nih.gov/protein/NP_000901.1)  [NP_001192028.1](https://www.ncbi.nlm.nih.gov/protein/NP_001192028.1)  [XP_020556937.2](https://www.ncbi.nlm.nih.gov/protein/XP_020556937.2)  [XP_021331562.1](https://www.ncbi.nlm.nih.gov/protein/XP_021331562.1)  [XP_013129547.2](https://www.ncbi.nlm.nih.gov/protein/XP_013129547.2)  [XP_021430896.1](https://www.ncbi.nlm.nih.gov/protein/XP_021430896.1)  [XP_030206371.1](https://www.ncbi.nlm.nih.gov/protein/XP_030206371.1)  [XP_044045802.1](https://www.ncbi.nlm.nih.gov/protein/XP_044045802.1)  [NP_001098693.1](https://www.ncbi.nlm.nih.gov/protein/NP_001098693.1)  [XP_051240664.1](https://www.ncbi.nlm.nih.gov/protein/XP_051240664.1)  [XP_020469972.1](https://www.ncbi.nlm.nih.gov/protein/XP_020469972.1)  [XP_015200024.1](https://www.ncbi.nlm.nih.gov/protein/XP_015200024.1)  [XP_019962041.1](https://www.ncbi.nlm.nih.gov/protein/XP_019962041.1)  [NP_001079348.1](https://www.ncbi.nlm.nih.gov/protein/NP_001079348.1) |
| *npy4r* | Human  Mouse  Nile tilapia  Torafugu  Chinese perch  Rainbow trou | [NP_001265723.1](https://www.ncbi.nlm.nih.gov/protein/NP_001265723.1)  [NP_032945.3](https://www.ncbi.nlm.nih.gov/protein/NP_032945.3)  [XP_005474155.1](https://www.ncbi.nlm.nih.gov/protein/XP_005474155.1)  [XP_029690445.1](https://www.ncbi.nlm.nih.gov/protein/XP_029690445.1)  [XP_044070308.1](https://www.ncbi.nlm.nih.gov/protein/XP_044070308.1)  [NP_571515.1](https://www.ncbi.nlm.nih.gov/protein/NP_571515.1) |
| *npy5r* | Human  Mouse | [NP_001304020.1](https://www.ncbi.nlm.nih.gov/protein/NP_001304020.1)  [NP_001345886.1](https://www.ncbi.nlm.nih.gov/protein/NP_001345886.1) |
| *npy6r* | Human  Mouse | [NP_001007219.1](https://www.ncbi.nlm.nih.gov/protein/NP_001007219.1)  [NP_035065.1](https://www.ncbi.nlm.nih.gov/protein/NP_035065.1) |
| *npy7r* | Chinese perch  Torafugu  Atlantic cod  Nile tilapia | [XP_044061435.1](https://www.ncbi.nlm.nih.gov/protein/XP_044061435.1)  [NP_001098695.1](https://www.ncbi.nlm.nih.gov/protein/NP_001098695.1)  [XP_030225112.1](https://www.ncbi.nlm.nih.gov/protein/XP_030225112.1)  [XP_005467973.1](https://www.ncbi.nlm.nih.gov/protein/XP_005467973.1) |
| *npy8ar* | Medaka  Zebrafish  Chinese perch  Rainbow trout  Nile tilapia  Torafugu | [XP_023816194.1](https://www.ncbi.nlm.nih.gov/protein/XP_023816194.1)  [NP_571512.1](https://www.ncbi.nlm.nih.gov/protein/NP_571512.1)  [XP_044030845.1](https://www.ncbi.nlm.nih.gov/protein/XP_044030845.1)  [XP_021446338.1](https://www.ncbi.nlm.nih.gov/protein/XP_021446338.1)  [XP_005458375.1](https://www.ncbi.nlm.nih.gov/protein/XP_005458375.1)  [XP_029692902.1](https://www.ncbi.nlm.nih.gov/protein/XP_029692902.1) |
| *npy8br* | Medaka  Zebrafish  Chinese perch  Nile tilapia  Torafugu | [XP_020561804.1](https://www.ncbi.nlm.nih.gov/protein/XP_020561804.1)  [NP_571511.1](https://www.ncbi.nlm.nih.gov/protein/NP_571511.1)  [XP_044053231.1](https://www.ncbi.nlm.nih.gov/protein/XP_044053231.1)  [XP_025752935.1](https://www.ncbi.nlm.nih.gov/protein/XP_025752935.1)  [NP_001098074.1](https://www.ncbi.nlm.nih.gov/protein/NP_001098074.1) |

## Supplementary Figures


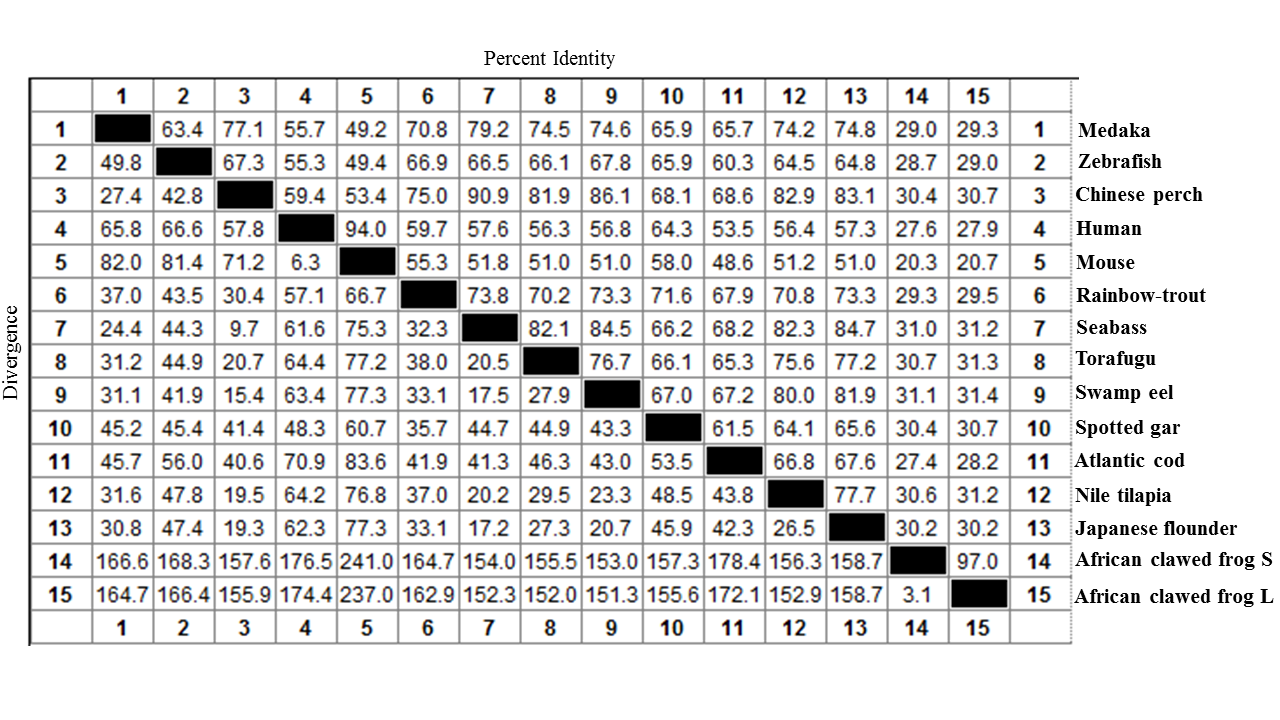


**Supplementary Figure 1.** Analysis of similarity and consistency of amino acid sequence of *npy2r* in medaka.
